# Reducing Maladaptive Behaviors in Preschool-Aged Children with Autism Spectrum Disorder Using the Early Start Denver Model

**DOI:** 10.3389/fped.2014.00040

**Published:** 2014-05-09

**Authors:** Elizabeth Fulton, Valsamma Eapen, Rudi Črnčec, Amelia Walter, Sally Rogers

**Affiliations:** ^1^KU Marcia Burgess Autism Specific Early Learning and Care Centre (ASELCC), Liverpool, NSW, Australia; ^2^Academic Unit of Child Psychiatry, School of Psychiatry, University of New South Wales, Sydney, NSW, Australia; ^3^Infant, Child and Adolescent Mental Health (ICAMHS), South Western Sydney Local Health District, Liverpool, NSW, Australia; ^4^School of Psychiatry and Behavioral Sciences, University of California Davis Medical Center MIND Institute, Davis, CA, USA

**Keywords:** autism spectrum disorder, Early Start Denver Model, maladaptive behavior, early intervention, efficacy

## Abstract

The presence of maladaptive behaviors in young people with autism spectrum disorder (ASD) can significantly limit engagement in treatment programs, as well as compromise future educational and vocational opportunities. This study aimed to explore whether the Early Start Denver Model (ESDM) treatment approach reduced maladaptive behaviors in preschool-aged children with ASD in a community-based long day care setting. The level of maladaptive behavior of 38 children with ASD was rated using an observation-based measure on three occasions during the intervention: on entry, 12 weeks post-entry, and on exit (post-intervention) over an average treatment duration of 11.8 months. Significant reductions were found in children’s maladaptive behaviors over the course of the intervention, with 68% of children showing a treatment response by 12 weeks and 79% on exit. This change was accompanied by improvement in children’s overall developmental level as assessed by the Mullen scales of early learning, but not by significant changes on the Vineland Adaptive Behavior Scales-II or Social Communication Questionnaire. Replication with a larger sample, control conditions, and additional measures of maladaptive behavior is necessary in order to determine the specific factors underlying these improvements; however, the findings of the present study suggest that the ESDM program may be effective in improving not only core developmental domains, but also decreasing maladaptive behaviors in preschool-aged children with ASD.

## Introduction

Autism spectrum disorder (ASD) is a life-long neurodevelopmental disorder characterized by impairments in social interaction and communication, and restricted, repetitive patterns of behavior, activities, or interests (1). The prevalence of ASD appears to be rising worldwide (2), with ASD estimated to affect around 1 in every 88 persons (3).

Autism spectrum disorder is recognized as a major public health concern because of its early onset, life-long persistence, and high levels of associated impairment (4). This impairment is attributable not only to the core symptoms of ASD, but also to the range of co-existing conditions that individuals with ASD often experience, including emotional and behavioral problems, sleep, feeding and eating problems, sensory sensitivities, learning and intellectual disabilities, as well as co-morbid health and mental health diagnoses (5). These co-existing conditions can be of equal or greater concern for parents and teachers of children with ASD than the core features of ASD, and have a significant impact on behavior management, learning acquisition, and the development of social relationships (6).

Problem behaviors (or maladaptive behaviors as they are referred to in this paper), characterized by disruptive, destructive, aggressive, or significantly repetitive behaviors, are prevalent in young children with ASD (7). For example, Dominick et al. (7) found that 32.7% of children with ASD displayed aggressive behaviors including hitting, kicking, biting, and pinching others. More than three-quarters of these children showed aggressive behaviors both at home and outside the home, and aggression was directed toward more than one person in 92% of cases. Self-injurious behavior, including head banging, hitting oneself, and biting oneself, was present in almost one-third of children with ASD (7). Furthermore, 70.9% of children with ASD had experienced a period of severe temper tantrums and, for 60% of these children, tantrums occurred on a daily basis and were a constant (rather than episodic) problem during the period in which they were present (7).

Several authors have noted a strong negative relationship between the ability to communicate and the prevalence of maladaptive behavior in young children with ASD (8). Self-injurious behaviors among children with ASD have also been linked to their receptive and/or expressive communication deficits (9). It follows that when treatment programs focus on developing the young child’s communication skills to the extent that they can serve as effective replacement behaviors, a reduction in the maladaptive behavior may result (10).

Maladaptive behaviors are particularly problematic in group settings, such as early intervention services, childcare services, and preschools, as they can be disruptive to the learning program and pose significant challenges to the children with ASD themselves, their peers, and staff. For these reasons, maladaptive behaviors are amongst the most commonly identified barriers to the inclusion of children with ASD in group settings (11).

Further, once maladaptive behaviors become an established part of a child’s behavioral repertoire, they are unlikely to decrease and, according to Berg et al. (12), will typically remain or worsen without intervention. If left untreated, these behaviors can significantly reduce a child’s social and educational opportunities by limiting their access to available treatments, learning activities, interactions with others, community experiences and, in particular, their ability to transition to, and participate in, school programs (13). In addition to having a negative impact on children with ASD themselves, a number of studies have shown that parents’ level of psychological distress is associated with the severity of their child’s maladaptive behaviors as well as their ASD symptoms (14–20).

Therefore, early interventions for young children with ASD should incorporate the management of maladaptive behaviors (21). Given the relationship between maladaptive behaviors and deficits in communication and social skills, it is important that intervention approaches target these core deficits. Myers and Johnson (22) argue that the primary goals of intervention for children with ASD should be to maximize the child’s functional independence and quality of life by reducing the core symptoms of ASD; facilitate development and learning; promote socialization; reduce maladaptive behaviors; educate and support families. They suggest that, in addition to targeting communication and social skills, contemporary comprehensive intervention approaches for ASD should target a reduction in disruptive or maladaptive behavior by using empirically supported strategies, including functional behavior assessment (FBA). FBA is “the process of determining the intent an inappropriate behavior serves for obtaining a desired outcome and replacing that behavior with a more appropriate one that accomplishes the same goal” [Ref. (23), 136 p.].

The general importance of early intervention for ASD is widely recognized, and is supported by studies showing better outcomes with earlier treatment (24, 25). Early intervention for ASD, especially that commencing before the age of 3 years, results in significantly improved outcomes relative to intervention commencing later in life (26–28). Early intervention in the first years of life offers the best potential for children as brain plasticity is greatest during this period, enabling the establishment and reorganization of neuronal networks in response to environmental stimulation (26).

A review of research conducted by Prior et al. (29) to identify the most effective models of early intervention for children with ASD classifies approaches into three main categories.

Each target maladaptive behaviors differently:

- Biologically based interventions, including medication, have been used to treat the co-morbid symptoms of ASD such as anxiety and hyperactive behavior with varying degrees of effectiveness.
- Psychodynamic interventions target the emotional component of behavior only. However, because ASD is considered a neurodevelopmental, rather than emotional, disorder, there is little empirical evidence demonstrating their effectiveness.
- Educational interventions including behavioral interventions such as applied behavior analysis (ABA); the Lovaas program; Pivotal Response Training; developmental and relationship-based interventions including ESDM; communication-focused interventions and sensory–motor interventions tend to have a positive treatment response. Each of these approaches uses different mechanisms to target maladaptive behaviors, and some advocate for the use of FBA as part of this process. These interventions usually approach behavior modification directly, focusing on the behavior itself.

Programs such as the Early Start Denver Model (ESDM) focus on building communication skills, especially by following the child’s lead and increasing the reinforcement value of social interaction, thereby teaching children adaptive ways of getting their needs met (30). Given that the ESDM is designed to enhance the social attention and communicative abilities of young children with ASD, with particular focus on the critical skills of social attention, affect sharing, imitation, and joint attention, it is conceivable that a significant reduction in maladaptive behavior may result.

Several meta-analyses conducted in recent years have tended to conclude that early intensive behavioral intervention (EIBI), generally defined as intervention that is delivered at an intensity of 15–20 h/week, incorporating the principles of ABA, is the treatment of choice for young children with ASD [cf. (8, 31)]. The literature indicates that superior outcomes are associated with entry into EIBI at the earliest possible age (32, 33).

The only comprehensive EIBI program available for children aged <30 months that has been empirically evaluated is the ESDM (27). The ESDM is specifically designed for children aged 12–60 months and is a manualized, comprehensive intervention that integrates ABA into a developmental and relationship-based approach (30). The ESDM is an intensive and comprehensive early intervention model that aims to reduce the severity of ASD symptoms and accelerate children’s development in all areas, with particular emphasis in the cognitive, social–emotional, and language domains.

The ESDM draws from teaching practices developed in the original Denver Model such as relationship-based aspects of the therapist’s work with the child, using play as a foundation for learning, and using communication intervention principles from the field of communication science (30). Positive behavior approaches focus on replacement of unwanted behaviors with more conventional behaviors and FBA is used when behaviors are more challenging.

The first and only randomized controlled trial of the ESDM demonstrated that, compared with children receiving community intervention, children receiving the ESDM showed significant gains in visual processing and improvements in language abilities, with subsequent gains in IQ and adaptive behaviors (27). In that study, children received 20 h/week of one-to-one ESDM intervention in a University clinic setting. There was also a separate parent training module. Two further studies (34, 35) have investigated the efficacy of delivery of the ESDM in group settings. Both studies reported significant developmental gains following the intervention and Eapen et al. (34) also found a significant decrease in autism-specific symptoms.

While Dawson et al. (27) and Eapen et al. (34) investigated the impact of the ESDM on children’s adaptive behavior, no studies of the ESDM to date have focused on the effect of the ESDM on children’s maladaptive behaviors. Given the adverse effect that maladaptive behaviors have on children with ASD, as well as their parents, it is important to investigate the impact of interventions on these behaviors. This was the primary aim of the present study.

## Materials and Methods

The study was approved by the local institutional and University ethics committees and all families recruited to the study provided informed consent to participate.

### Study design and participants

A pre–post study of children treated with ESDM was conducted. Note that clinical outcomes, but not ESDM clinician behavioral ratings data, for a portion of the cohort have been described previously in Eapen et al. (34). Participants were 38 children attending an Autism Specific Early Learning and Care Centre (ASELCC) in metropolitan Sydney, Australia. The center is one of the six ASELCCs established by the Australian Government within the setting of a long day child care center for children aged 2–6 years. All children had a DSM-IV-TR diagnosis of Autistic Disorder, made by a community-based physician, with the exception of one child who had a diagnosis of pervasive developmental disorder not otherwise specified. These children would all have met criteria for a DSM-5 diagnosis of ASD. Exclusion criteria included neurological (e.g., uncontrolled epilepsy) disorders, and significant vision, hearing, motor, or physical impairment.

The average age of children at the time of study commencement was 52.2 months (SD 5.4, range: 38.8–63.7 months), and 35 (92%) were male. English was the primary language spoken at home in 82% of families, although 60% of families reported a cultural background other than Australian.

None of the participants were receiving an EIBI outside of the ESDM intervention offered as part of this program. No families withdrew from the study during the course of the intervention; however, there were instances of missing data due to families not completing measures within the necessary timeframes.

### Intervention

The study employed the ESDM curriculum and teaching principles within a group setting. Other than accommodations to allow translation to the group context, which are described in Eapen et al. (34), no modifications were made to the ESDM curriculum.

Rogers and Dawson (30) outline a specific teaching approach in the ESDM that was followed in this study. ESDM teaching principles are embedded in play and in natural daily routines within elaborated joint activity routines that address multiple objectives across multiple developmental domains. The main focus is on teaching imitation; developing awareness of social interactions and reciprocity; teaching the power of communication; teaching more flexible, conventional, and creative play skills; making the social world as understandable as the world of objects. Rogers and Dawson (30) contend that just as typically developing children spend their waking hours engaged in the social milieu and learning from it, children with ASD need to be drawn into a carefully prepared and planned social milieu that they can understand, predict, and participate in.

Whilst a primary focus on maladaptive behaviors is not central to the ESDM curriculum, the general approach in this model for children whose level of maladaptive behavior has not improved after 3 weeks of intervention follows the principles of positive behavior supports (36, 37). This is a method of applying the principles of ABA that focus on the use of reinforcement strategies to teach children adaptive and conventional behaviors for meeting their needs and expressing their feelings, as well as promoting independent functioning (30). There were only two children in the current sample whose behavior had not improved after 3 weeks of intervention. For these children, FBA was conducted by the Behavior Analyst on the Intervention team. This process determined the functions of the child’s behavior and the consequence that was reinforcing the behavior. This is based on the premise that the behavior is in the child’s current repertoire because it leads to a rewarding consequence; therefore the effects of a range of consequences must be tested to determine how the behavior is being reinforced or maintained. The FBA also enabled the Behavior Analyst to identify replacement behaviors that would serve a similar function for the child, but were more conventional behaviors for the child to learn. The ESDM then identifies the skills that can be converted into objectives and targeted in the child’s individualized program, so that he/she can quickly learn, master, and generalize the new adaptive behaviors to become part of their behavioral repertoire.

During their attendance at the center, participants received an hour of intensive individualized ESDM therapy each week, in addition to an hour of intensive small group ESDM therapy daily, and ESDM-driven learning experiences throughout the day. Each child also received between 15 and 20 h/week of group ESDM intervention. The one-to-one sessions were conducted by the child’s key worker, who carried a caseload of five-to-six children across the period of the intervention. Each child had an individualized treatment plan that incorporated a range of objectives dependent on the child’s level of functioning. These objectives were developed from the child’s initial assessment using the ESDM curriculum checklist, which includes a list of skills spanning receptive communication; expressive communication; social skills; joint attention behaviors; fine motor; gross motor; imitation; cognition; play skills; behavior; and personal independence (eating, dressing, grooming, and chores).

All interventions were delivered by therapists with tertiary-level qualifications who were trained to certification in the ESDM by an accredited trainer. In order to be certified in direct delivery of this model, therapists were required to achieve: (1) a fidelity rate of 80% or more with the ESDM trainer on each of the 13 ESDM teaching principles across multiple children and sessions, and (2) to achieve the same level of concordance on the individualized written treatment plans they had developed and data they collated on each child. That is, 80% or more concordance was required in both the clinical delivery and data recording aspects of the ESDM, including on the ESDM behavior checklist, which formed a key measure in this study. There were six key workers, each trained in this way, involved in the study. Therapists also continued to receive clinical supervision in their delivery of the ESDM by an Accredited Trainer.

### Measures

Pre- and post-measures included the (1) ESDM behavior rating as well as the (2) Vineland Adaptive Behavior Scales, second edition (Parent Form) [VABS-II; (38)]; (3) Social Communication Questionnaire [SCQ; (39)]; and (4) Mullen Scales of Early Learning [MSEL; (40)]. The ESDM behavior rating was also completed 12 weeks after entry to the program.

The rating of children’s maladaptive behavior was completed during the child’s individual 1 h ESDM session using the ESDM Behavior Coding system. The coding system allows therapists to quantify the child’s behavior for each 15-min period, as well as for the hourly session as a whole. The rating for the session as a whole was used in this study. This rating measured the level of maladaptive behavior that was typically present over the hour rather than the best or worst behavior observed during the session.

The Behavior Coding system designed by Rogers and Dawson (30) for measurement of maladaptive behaviors is described below:

1. *Severe problem behaviors* including aggression, self-injurious behavior, frequent and intense tantrums;
2. *Mild problem behaviors* including non-compliance, some tantrums, but able to participate to some extent;
3. *Some problem behaviors* including fussy, whining, some non-compliance, but able to participate in most of the activity;
4. *No problem behavior* but difficulty staying on task;
5. *Compliant* on task, working at ability level;
6. *Above average* performance for that child; pleasant, excited about the activity.

Rating of behavior codes was completed by each child’s key worker who conducted their individual ESDM therapy and was responsible for collecting their data within the group program also. These data were then discussed and peer reviewed in daily Key Worker meetings. Discrepancies were discussed with the ESDM trainer. Senior ESDM trainers working in the UC Davis MIND Institute were available to discuss significant discrepancies; however this was not required for any behavioral ratings. All child data, including the behavior codes, were reviewed on a quarterly basis by the ESDM trainer, including through the use of videos of therapy sessions or live viewing (from the observation room) of therapy sessions to ensure ongoing fidelity.

A pre-intervention behavior score was coded on entry to the program (in the therapy session following the initial assessment), a second behavior score was coded after the first 12 weeks of intervention, and a post-intervention behavior score was coded before the child exited the program.

Parents of participating children completed two measures. The VABS-II (38) assesses parents’ perceptions of their child’s everyday adaptive functioning in the domains of Communication (including expressive and receptive language), Daily Living Skills, Socialization, and Motor Skills. For each domain, including an overall Adaptive Behavior Composite, a norm-referenced standardized score with a mean of 100 and SD of 15 is calculated. *V* -scale scores with a mean of 15 and a SD of 3 and age-equivalent scores are calculated for each sub-domain, including Internalizing Behavior, Externalizing Behavior, and the Maladaptive Behavior Index. The VABS-II has well-established strong psychometric properties (38). The SCQ (39) is a 40-item measure of autism-specific symptoms where scores of 15 or more indicate probable ASD. The SCQ has robust psychometric properties (41–43). These measures were administered at two time points (on entry to and exit from the program). Parents also completed a demographic questionnaire at the start of the study.

In addition, children were assessed at entry to and exit from the program using the MSEL (40), a widely used, standardized measure of early development for children aged from birth to 68 months, yielding standardized *T* Scores and age-equivalent scores on the following subscales: Visual Reception, Fine Motor, Receptive Language, Expressive Language, and Gross Motor. The Gross Motor subscale was not administered in this study. Given the majority of children in the current sample did not receive MSEL subscale raw scores that were high enough for calculation of a meaningful *T* score (i.e., they were performing at a level <0.1 percentile), standardized developmental quotients (DQs) were calculated for each subscale of the MSEL by dividing each child’s age-equivalent score by their chronological age at the time of testing and multiplying by 100, as is common practice. In this regard, a child who was aged 48 months, but who had an age-equivalent score of 24 months, would receive a DQ of (24/48) × 100 = 50. An overall DQ was also calculated for each child by taking the average of the child’s DQs for the four completed subscales in order to provide an estimate of overall intellectual ability. Note that the sum of the *T* scores for these four subscales (i.e., Visual Reception, Fine Motor, Receptive Language, and Expressive Language) is used to calculate the Early Learning Composite Score of the MSEL. It should also be noted that the DQs calculated in this study are not equivalent to *T* scores or the Early Learning Composite Score of the MSEL, but represent an attempt by the study team to standardize scores for the purpose of making comparisons over time.

### Statistical analysis

Paired samples *t*-tests were conducted to compare children’s scores pre- and post-intervention on the aggregate measures of clinician ESDM child behavior ratings; Vineland Adaptive Behavior Composite score; Vineland Maladaptive Behavior Index Score; SCQ total score; and overall MSEL DQ. Cohen’s *d* effect sizes were also reported. It is widely accepted that Cohen’s *d* values of 0.2–0.49 denote small-sized effects; 0.5–0.79 denote medium-sized effects; and >0.8 denote large effect sizes. To explore change, pre- and post-intervention in the subscales of measures used, a series of repeated measures MANOVA analyses were conducted using the Pillai’s Trace criterion. The aggregate scores noted above were not included in these MANOVA analyses as these scores were not independent of the subscale scores. Partial eta values were reported as a measure of effect size for MANOVA analyses. Correlations were also computed to investigate relationships between children’s behavior and baseline demographic and clinical variables. Analyses were conducted using SPSS statistical software. Alpha was set at 0.05 for the majority comparisons, following recommendations by Saville (44) who argues for this per-comparison level rather than a family wise approach when conducting research in novel areas. An exception to this was in the instance where multivariate effects detected in the MANOVA analyses were further explored using paired samples *t*-tests. In those cases, a Bonferroni correction was applied.

## Results

The average time between pre- and post-intervention assessment was 11.8 months (SD 5.8). As shown in Table 1, a significant reduction in clinician-rated ESDM behavior rating was found, *t*(37) = −16.6, *p* < 0.001. The size of this effect was Cohen’s *d* = −3.7, which is large. There was also a significant increase to children’s overall MSEL DQ, *t*(17) = −5.0, *p* < 0.001, *d* = −0.41, which approaches a medium-sized effect. There was, however, no significant change in children’s VABS-II Adaptive Behavior Composite, VABS-II Maladaptive Behavior Index, or SCQ total scores.

**Table 1:** **Pre- to post-intervention scores in a cohort of preschoolers treated with group ESDM**.

	Pre-intervention		Post-intervention		*t*^a^	df	*p*	Cohen’s *d*^h^
	Mean	SD	Mean	SD	
ESDM behavior rating	1.8	1.0	5.1	0.8	−16.6	37	<0.001**	− 3.67
**VINELAND ADAPTIVE BEHAVIOR SCALES-II STANDARD DOMAIN SCORES**								
Communication^b^	62.4	15.2	64.8	19.7				− 0.14
Socialization^b^	66.8	14.2	63.7	13.6				0.22
Daily Living Skills^b^	62.1	14.7	62.2	16.6				− 0.01
Motor Skills^b^	69.4	20.7	65.3	23.2				0.19
Adaptive Behavior Composite^b^	62.2	14.8	62.5	14.7	−0.2	12	0.84	− 0.02
**VINELAND ADAPTIVE BEHAVIOR SCALES-II MALADAPTIVE BEHAVIOR**								
Internalizing Behavior^c^	19.4	1.8	18.9	4.0	0.5	13	0.60	0.17
Externalizing Behavior^c^	16.0	2.2	15.1	3.0	1.0	13	0.34	0.35
Maladaptive Behavior Index^c^	18.8	1.4	18.8	1.8	0.0	13	1.0	0.00
SCQ total score^d^	18.3	6.3	17.0	7.3	1.0	13	0.34	0.19
**MULLEN SCALES OF EARLY LEARNING**								
Visual Reception DQ^e^	37.2	19.9	48.3	27.3	−2.7	19	0.013^g^^,^*	− 0.47
Fine Motor DQ^e^	46.3	24.3	50.6	21.2	−1.4	21	0.17^g^	− 0.19
Receptive Language DQ^e^	30.4	22.3	39.7	24.4	−3.5	17	0.003^g^^,^**	− 0.40
Expressive Language DQ^e^	33.4	18.4	40.7	20.0	−4.5	20	<0.001^g^^,^**	− 0.38
Overall MSEL DQ^f^	37.9	19.8	46.5	22.2	−5.0	17	<0.001^g^^,^**	− 0.41

*p < 0.05, **p < 0.01, SCQ, Social Communication Questionnaire.

*For the SCQ total score, lower scores are indicative of fewer ASD symptoms. Similarly, for VABS-II Maladaptive Behavior subscales (Internalizing Behavior, Externalizing Behavior, and Maladaptive Behavior Index), lower scores denote fewer symptoms. For all other measures, higher scores are indicative of better functioning*.

*^a^Paired samples *t*-tests were conducted *a priori* for aggregate scores of ESDM behavior rating, VABS-II Adaptive Behavior Composite, VABS-II Maladaptive Behavior Index, SCQ total and overall MSEL DQ. In other instances, paired samples *t*-tests were conducted only following significant results in multivariate repeated measures MANOVA analyses*.

*^b^Standard score (mean: 100, SD: 15)*.

*^c^*V*-scale score (mean: 15, SD: 3)*.

*^d^Range = 0–40. Scores of 15 or more denote probable ASD*.

*^e^DQ (developmental quotient) = (age-equivalent score/chronological age) × 100*.

*^f^Overall MSEL DQ = (Visual Reception DQ + Fine Motor DQ + Receptive Language DQ + Expressive Language DQ)/4*.

*^g^Bonferroni adjusted α = 0.013*.

*^h^Following the recommendations of Dunlap et al. (45), Cohen’s *d* scores were calculated using the pooled standard deviation score uncorrected for the correlation between pre-post scores*.

To explore changes in core subscales of the VABS-II, a repeated measures MANOVA was performed with VABS-II standard domain scores as the dependent variables (Communication; Socialization; Daily Living Skills; and Motor Skills). The within-subjects independent variable was time, with two levels (pre-intervention and post-intervention). There was no significant multivariate effect of time *F*(1, 11) = 0.18, *p* < 0.05 or VABS-II subscale scores *F*(3, 9) = 2.8 *p* > 0.05, nor a domain scores by time interaction. With respect to the VABS-II Maladaptive Behavior subscales, a repeated measures MANOVA was performed with Internalizing Behavior and Externalizing Behavior as the dependent variables and time as the within-subjects independent variable. The multivariate effect of time *F*(1, 13) = 0.67, *p* < 0.001 and the time by VABS-II Maladaptive Behavior subscale score interaction *F*(1, 13) = 0.18, *p* > 0.05 were not statistically significant. However, the multivariate effect for subscale scores was significant *F*(1, 13) = 23.1, *p* < 0.001, ${\eta_{p}}^{2}=0.64$. When explored further using paired sample *t*-tests with an adjusted alpha rate of 0.05/2 = 0.025 neither of the Internalizing or Externalizing scores changed significantly over time, however effect sizes were non-trivial (see Table 1). In the case of the MSEL, a repeated measures MANOVA was performed with Visual Reception DQ, Fine Motor DQ, Receptive Language DQ, and Expressive Language DQ as the dependent variables and time as the within-subjects independent variable. The multivariate effects of MSEL subscale scores *F*(3, 15) = 6.5, *p* = 0.005, ${\eta_{p}}^{2}=0.57$ and time *F*(1, 17) = 24.69, *p* < 0.001, ${\eta_{p}}^{2}=0.59$ were significant; however, the subscale scores by time interaction were not. When explored further using paired sample *t*-tests with an adjusted alpha rate of 0.05/4 = 0.013, the Visual Reception DQ, Receptive Language DQ, and Expressive Language DQ all showed significant improvement from pre- to post-intervention with effect sizes approaching medium size (see Table 1).

To further explore the speed with which improvement in the level of maladaptive behaviors occurred, *post hoc* analyses were conducted using ESDM clinician-rated behavior checklist data obtained at entry, 12 weeks post-entry, and exit from the ESDM program. It emerged that, at entry to the program, only one of the 38 children had a behavior score of 5 or 6 (indicating compliant or above average behavior). This number increased to 26 of 38 children (68%) after 12 weeks of intervention, and to 30 of 38 children (79%) by the end of the intervention.

A related analysis involved examining the number of participants whose scores improved by three points or more on the six point scale (taken to denote a conservative estimate of meaningful change) at the different time points. One participant had an entry score that would preclude improvement by three points; hence subsequent analyses were conducted on the remaining 37 children. After 12 weeks of intervention, 25/37 children (68%) had improved by three or more points (rapid responder sub-group), whereas 32% of children had not responded in this way (non-responder sub-group). By program exit, the non-responder group had dropped to 24% of the sample.

A series of independent samples *t*-tests were conducted to examine whether the rapid responder sub-group differed from the 12-week non-responder sub-group according to baseline variables. Given the relatively small sample size in these analyses, MANOVAs were not performed and Cohen’s *d* effect sizes were also inspected for cases where the effect size was of medium size or larger (Cohen’s *d* > 0.50). Analysis revealed that the SCQ score for the rapid responder sub-group (mean = 16.1) was lower than that of the non-responder sub-group (mean = 21.5) at a level that approached significance *t*(27) = 1.86, *p* = 0.07, Cohen’s *d* = 0.77, that is, the rapid responder group tended to have lower baseline ASD symptoms than the non-responder group. Other areas where the difference between rapid responder and non-responder groups was above Cohen’s *d* = 0.5 at baseline were VABS-II Communication, Daily Living Skills, and Motor Skills Standard Scores. In all instances, the rapid responder group performed better than the non-responder group at baseline.

Correlations between baseline clinical variables and pre- and post-intervention behavior ratings as well as change in behavior ratings are presented in Table 2. As shown in Table 2, clinician-rated behavior at entry was not significantly related to any baseline clinical variables, including DQs, autism severity, or adaptive behavior (all *p*s > 0.05).

**Table 2:** **Correlations between clinician-rated behavior scores and baseline clinical variables**.

	Entry behavior	Exit behavior	Change in behavior
Entry behavior rating	–	–	–
Exit behavior rating	0.14	–	–
Change in behavior rating	−0.72**	0.59**	–
VABS-II Communication	0.22	0.26	−0.04
VABS-II Socialization	0.22	0.14	−0.12
VABS-II Daily Living Skills	0.25	0.42*	−0.02
VABS-II Motor Skills	0.12	0.36	0.10
VABS-II ABC	−0.23	0.31	−0.02
VABS-II Internalizing	−0.17	−0.09	0.08
VABS-II Externalizing	−0.05	0.37*	0.28
VABS-II Maladaptive	−0.13	0.02	0.10
SCQ total	0.08	−0.20	−0.20
SCQ Communication	0.16	−0.18	−0.26
SCQ Restricted Social Interaction	−0.13	−0.24	−0.06
SCQ Repetitive Behavior	0.13	−0.09	−0.17
VRDQ	0.07	0.37	0.18
FMDQ	0.17	0.55**	0.21
RLDQ	0.26	0.48*	0.10
ELDQ	0.32	0.46*	0.02
Overall DQ	0.23	0.53**	0.16

*p < 0.05, **p < 0.01, VABS-II, Vineland Adaptive Behavior Scales-II; ABC, Adaptive Behavior Composite; SCQ, Social Communication Questionnaire; VRDQ, Visual Reception DQ Score; FMDQ, Fine Motor DQ Score; RLDQ, Receptive Language DQ Score; ELDQ, Expressive Language DQ Score.

Clinician-rated behavior at exit was shown to be significantly and positively correlated with Fine Motor, Receptive Language, Expressive Language, and overall DQs at entry (*r* = 0.46–0.55, *p*s < 0.05). Clinician-rated behavior at exit was also positively correlated with baseline daily living skills (*r* = 0.42, *p* < 0.05), that is, the better a child’s daily living skills at entry, the better their clinician-rated behavior at exit. Finally, clinician-rated behavior at exit was found to be positively correlated with baseline externalizing behavior, as measured by standardized VABS-II scores (*r* = 0.37, *p* < 0.05), suggesting that the more problematic a child’s externalizing behavior at entry, the better their clinician-rated behavior at exit.

Change in clinician-rated behavior was not found to be significantly associated with any baseline variables (all *p*s > 0.05).

## Discussion

Children with ASD frequently engage in maladaptive behaviors such as aggression, self-injurious behavior, and stereotyped behaviors (7). These behaviors are problematic in group settings, as they disrupt the learning program and place children at increased risk for social exclusion, making it very difficult for them to transition to and access mainstream education settings (13). These behaviors also correlate positively with levels of stress in caregivers (17). While the genesis of these maladaptive behaviors is thought principally to reside in communication and social skills difficulties, there is some uncertainty in the literature as to whether maladaptive behaviors are best managed via direct behavioral intervention; via treatments targeted primarily at improving pro-social and communicative skills; or via a combination approach. This study sought to examine the behavioral benefits to maladaptive behaviors of the ESDM, an early intervention focused predominantly on improving communication and pro-social skills, within natural daily play and care routines.

Several key findings were obtained. Principally, the level of maladaptive behaviors in the cohort of children studied, as assessed by clinician rating, reduced substantially following the 11-month ESDM intervention period. Moreover, for 68% of the children studied, substantial positive change was observed within the first 12 weeks of intervention. This group, who we have described as “rapid responders” tended to have less severe ASD symptoms at baseline and had a higher level of communication, daily living, and motor skills at baseline compared with children whose level of maladaptive behavior did not respond quickly to the ESDM. The behavior rating obtained at entry was not associated with any of the other baseline variables, which together with the finding that only 1 out of the 38 participants had ratings of good behavior at baseline, suggests that maladaptive behaviors occurred relatively uniformly within the sample. Across the whole sample, the degree of change in behavior rating from pre- to post-intervention was not associated with any baseline variables. However, clinician-rated behavior at exit was shown to be significantly and positively correlated with Fine Motor, Receptive Language, Expressive Language, and overall DQs; daily living skills; higher level of externalizing behavior at entry. In general terms, we would contend therefore that while maladaptive behaviors appear to have been ubiquitous in our cohort, children with relatively better adaptive functioning and fewer ASD symptoms at baseline seemed more likely to show rapid and subsequent improvement in their level of maladaptive behaviors. Overall, however, more than three-quarters of participants showed improvements (of three points on the six point scale) in maladaptive behaviors by the end of the intervention. Given the negative consequences of maladaptive behavior on children’s learning (6), the ESDM’s ability to bring about reductions in maladaptive behaviors – early in the intervention for around 3/4 of participants – may have allowed children to access and gain from the intervention program more effectively.

Significant improvements were also found following ESDM intervention in MSEL Visual Reception, Receptive Language, Expressive Language, and overall DQs. This is consistent with previous research (27, 34, 35). It is possible that, by promoting child development across domains, particularly receptive and expressive communication, and by using appropriate behavior management strategies, the ESDM resulted in an increase in conventional behaviors and a reduction of maladaptive behaviors. This is consistent with research showing a strong relationship between communication skills and the presence of maladaptive behavior in young children with ASD (8), and provides support for the suggestion by Myers and Johnson (22) that contemporary comprehensive intervention approaches for ASD should target communication and social skills in addition to disruptive or maladaptive behavior.

Furthermore, a child who is highly motivated is also more likely to learn at a faster rate (8). The ESDM works to increase child motivation by incorporating components such as child choice, turn taking, reinforcing attempts, and interspersing maintenance with acquisition tasks (30). The ESDM therapist is also highly trained in managing child attention; delivering clear antecedent, behavior, consequence sequences; modulating child arousal; creating interesting routines; building dyadic engagement though joint activity routines; responding with sensitivity to all child communicative attempts. The teaching principle that targets modulation of child arousal equips ESDM therapists to recognize and respond immediately to changes in child arousal levels and modulate these in the moment, potentially preventing maladaptive behaviors from developing in the first place.

Despite improvements in clinician-rated behavior and developmental skills, maladaptive behavior ratings on the VABS-II did not show improvement from pre- to post-intervention. It is interesting to observe that the externalizing behavior score on the VABS-II did show the largest Cohen’s *d* effect size change of any VABS-II score (*d* = 0.35), but this was not statistically significant. One possible explanation for this finding is offered by Weiss et al. (46), who question the validity of the Maladaptive Behavior domain of the VABS-II in assessing levels of maladaptive behavior among children with ASD. It is also important to note that normative data on the VABS are only available for much older children than those in the current sample, and is not available for those with ASD. It is also possible that, while children’s behavior during ESDM therapy sessions improved, this improvement did not generalize to the home environment and therefore no changes were found in parent-reported maladaptive behavior. Mastering the teaching principles of the ESDM equips adults to engage, modulate, and motivate the child into an optimal state for learning, hence promoting pro-social behavior. It is possible that the optimal behavior elicited during ESDM sessions was not replicated in other settings as parents or other caregivers were not similarly equipped with the skill set to elicit these pro-social behaviors. This suggestion highlights the potential importance of training parents and other professionals, such as those in school settings, in the ESDM model in order to provide the child adequate opportunities to generalize their newly acquired skills, and ideally of future research to explore the relative outcome for children in groups where parents had, or had not received intervention. We note that there was no specific parent training component to the ESDM intervention applied in this study; however, optional parent education evenings were offered at the center. Similarly, no significant improvements were found in the VABS-II standard domain scores or on the SCQ. This could again be attributable to these measures being parent reports, and skills not generalizing to the home setting; however, the lack of change observed in the current study on the SCQ is inconsistent with findings of significant improvement on this measure by Eapen et al. (34).

Findings of reduced clinician-rated maladaptive behavior and accelerated developmental rates in the present study are promising; however, due to the design of the current study, it is not possible to make conclusions about the mechanisms behind these improvements. That is, it is not possible to determine whether the reduction in maladaptive behavior observed in the present study was a consequence of the ESDM’s focus on social attention, affect sharing, imitation, and joint attention, or whether it was due to the use of behavioral techniques that are not specific to the ESDM, such as FBA and positive behavior supports, which have previously been shown to be effective in managing behavior within the framework of multiple treatment approaches. While ABA principles, FBA, and positive behavior supports are integral components of the ESDM, it is important to note that their specific implementation was only required for 2 of the 38 children in the present study whose behavior had not significantly improved after 3 weeks of intervention. It is therefore unlikely that the improvements in maladaptive behavior observed in the present study were directly and solely attributable to these behavioral strategies. Nonetheless, it is necessary to replicate the present study using a larger cohort and control conditions (both a different treatment condition and a non-treatment condition) in order to establish whether the reductions in maladaptive behavior occurring during ESDM intervention are significantly different to reductions that may occur in the context of a different treatment program or by maturation alone.

Regardless of the exact mechanisms behind the improvements in maladaptive behavior in the present study, our findings suggest that the ESDM program may be an effective tool in improving not only core developmental domains, but also decreasing maladaptive behaviors in preschool-aged children. This finding is important, given previous research demonstrating the negative impact of maladaptive behaviors and developmental delays on the child’s learning acquisition and the development of social relationships (6). The relatively quick reduction in maladaptive behaviors observed in the present study (68% of children showed a significant decrease in maladaptive behavior by 12 weeks) may allow children to more effectively participate in and benefit from learning opportunities, including the intervention itself, and may be a key factor in the developmental gains observed in the present study and previous research (27, 34, 35). It is hypothesized that these developmental gains, particularly in the areas of receptive and expressive communication, may then provide children with adaptive means of getting their needs met, thereby further reducing maladaptive behaviors.

### Limitations

This pilot study was limited by the use of a clinician-rated behavior score as the main dependent variable, particularly given that there were no blind raters on any measures. The fact that significant improvements were found in this rating over the course of the intervention, despite no change in VABS-II Internalizing, Externalizing, or Overall Maladaptive Behavior, raises questions over the reliability and validity of the ESDM clinician-rated behavior score. However, as noted previously, the validity of the VABS-II in assessing levels of maladaptive behavior among children with ASD has been questioned (46). Furthermore, the achievement of inter-rater reliability is fundamental to becoming certified as an ESDM therapist, with a requirement of initial and ongoing consistency of ratings with peers and the ESDM trainer. The fact that 32% of children did not show a change of three or more points in clinician-rated behavior over the first 12 weeks of the intervention is also an argument against rater bias.

A further limitation of the present study was the lack of a control group, which makes it difficult to determine whether the observed behavioral and developmental improvements were the effect of maturation or the intervention. Literature suggests, however, that maladaptive behaviors, once they become part of a child’s behavioral repertoire, will typically remain or worsen without intervention (12). Moreover, the size of the improvement in maladaptive behaviors observed was large *d* = 3.67, which suggests that maturation alone is unlikely to be the causative factor. Similarly, the common course among children with severe ASD presentations without intervention is for IQ to remain the same or regress (47). The children in the current study had relatively severe presentations, including MSEL DQs <47 and VABS-II adaptive behavior scores within the range of 62–70 at baseline. Therefore, it appears that the behavioral and developmental improvements observed from pre- to post-intervention in this study are unlikely to arise as a result of maturation. The uncontrolled design of the present study also means that it is not possible to determine whether the observed reductions in maladaptive behavior were the result of ESDM-specific principles or to behavioral techniques that are not specific to the ESDM. Therefore, replication of the present study using a larger cohort and control conditions is necessary. Follow-up studies are also required to determine whether the behavioral and developmental improvements observed in the present study are maintained, which has the potential to foster ongoing educational opportunities and improve quality of life for children with ASD and their families.

Since maladaptive and challenging behaviors often pose a barrier to inclusion and community participation with significant consequences on social and educational opportunities, harm or injury to self or others, and family distress, it is critical to address these behaviors in the comprehensive management of children with ASD. The findings of the present study are promising, suggesting that the ESDM delivered in a community setting with relatively minimal one-to-one intensive therapy has the potential to reduce children’s maladaptive behaviors which, in turn, may increase their capacity to participate in intervention and educational programs and make gains in other developmental domains.

## Conflict of Interest Statement

The authors declare that the research was conducted in the absence of any commercial or financial relationships that could be construed as a potential conflict of interest.
